# Association of Metabolically Healthy Obesity and Glomerular Filtration Rate among Male Steelworkers in North China

**DOI:** 10.3390/ijerph191811764

**Published:** 2022-09-18

**Authors:** Miao Yu, Shengkui Zhang, Lihua Wang, Hongman Feng, Xiaoming Li, Jianhui Wu, Juxiang Yuan

**Affiliations:** 1 Department of Epidemiology and Health Statistics, School of Public Health, North China University of Science and Technology, Tangshan 063210, China; 2 Department of Epidemiology and Statistics, Institute of Basic Medical Sciences, Chinese Academy of Medical, Beijing 100005, China

**Keywords:** metabolically healthy obesity, renal function, inflammation

## Abstract

This study aims to investigate the association between metabolically healthy obesity (MHO) and the early stages of renal dysfunction and whether systemic inflammation affects the study’s outcome. Male steelworkers in northern China were investigated in this cross-sectional survey (*n* = 6309). A decrease in estimated glomerular filtration rate (eGFR) was used as the primary outcome, which was defined as an eGFR of ≤89 mL/min/1.73 m^2^. A BMI ≥ 25 kg/m^2^ was used to determine obesity. In the definition of metabolic health, the absence of metabolic syndrome components is considered metabolically healthy. An assessment of inflammation was carried out using a surrogate marker called high-sensitivity C-reactive protein (hs-CRP). The adjusted odds ratio (OR) and confidence intervals (CIs) were estimated using the multivariable logistic regression model. After adjusting for hs-CRP, MHO (OR = 1.97; 95% CI: 1.21 to 3.21) was significantly associated with decreased eGFR compared to metabolically healthy non-obesity (MHNO). With the MHNO/hs-CRP ≤ 0.01 mg/dL group as a reference, the OR was 2.17 (95% CI: 1.17 to 4.02) for decreased eGFR in the group with MHO/hs-CRP > 0.01 mg/dL. MHO is associated with renal dysfunction at an early stage. To some degree, this risk can be explained by the level of inflammation.

## 1. Introduction

It is estimated that in 20 years, chronic kidney disease (CKD) will be the world’s fifth leading cause of death [1]. Approximately 10% of adults worldwide are affected by some form of CKD, which contributes to an elevated risk of end-stage renal disease (ESRD) and cardiovascular disease [2,3]. It is estimated that 11.6% of the Chinese population suffers from chronic kidney disease [4]. In the absence of an effective treatment for ESRD, it is essential to identify the risk factors of renal dysfunction at an early stage, which manifests as a mild decrease in estimated glomerular filtration rate (eGFR).

As is widely known, obesity is a global epidemic disease, and its prevalence rate has been rising since 1980 [5]. Approximately 46% of adults and 15% of children in China are overweight or obese [6]. Obesity is often associated with components of metabolic syndrome; a state often referred to as metabolically unhealthy obesity (MUO) [7]. Nevertheless, not all individuals with obesity develop metabolic abnormalities, and this group has been referred to as “metabolically healthy obesity (MHO)” [8]. Study participants are classified into four obesity phenotypes according to their obesity and metabolic status: metabolically healthy non-obesity (MHNO), metabolically unhealthy non-obesity (MUNO), MHO, and MUO. This definition of obesity phenotypes has often been used in global populations [9,10,11]. Currently, the relationship between different obesity phenotypes and health is still unclear, and experts have debated the existence of healthy obesity. It has been shown in an increasing number of studies that patients with MHO are at a higher risk of developing CKD than patients with MHNO, while a study in Japan found no increased risk [12,13,14]. In summary, it is not clear whether MHO is associated with the early stages of CKD.

Despite this, inflammation is thought to be one of the key factors explaining the metabolic changes associated with obesity. A study has shown that inflammation is an important factor contributing to obesity-related metabolic changes [15]. In addition, both obesity and metabolic abnormalities are associated with chronic subacute systemic inflammatory states, such as circulating pro-inflammatory cytokines and c-reactive proteins. Several studies have also reported an association between inflammation and CKD [16,17]. Based on these findings, we hypothesized that inflammation is a potential link between CKD and different obesity phenotypes. A better understanding of the link between MHO, inflammation, and mildly reduced eGFR is necessary and may have implications for predicting those susceptible to obesity-related complications, including CKD.

Furthermore, this will provide healthcare professionals with more accurate information to predict and intervene in early renal dysfunction. Currently, no large-scale study has been conducted to investigate this relationship. This study aims to investigate the association between metabolically healthy obesity (MHO) and the early stages of renal dysfunction, and whether inflammation affects this.

## 2. Materials and Methods

### 2.1. Study Populations

In this cross-sectional study, results of a baseline survey of steelworkers in 11 steel production sectors of the Tangsteel Company of the HBIS Group in North China are reported. Given that the proportion of female workers is less than 10% (7.9%) and that the jobs are very different from those of male workers, the current study only included male workers. A total of 7661 participants were recruited from February to June 2017. After excluding 603 female workers, 337 participants with no body mass index (BMI), 43 participants with no serum creatinine, and 369 participants with incomplete information on covariates, 6309 participants were ultimately included in this cross-sectional study. Ethics approval was granted by the North China University of Science and Technology’s ethics committee (Ethic ID: 16040). Informed consent forms were signed by all participants before taking part in the study.

### 2.2. Definitions

The Chronic Kidney Disease Epidemiology Collaboration (CKD-EPI) equation was used to estimate glomerular filtration rate (GFR) [18]. According to the Kidney Disease Improving Global Outcomes 2012 recommendations, a GFR between 60 and 89 mL/min/1.73 m^2^ is defined as mildly decreased renal function [19]. Considering only 19 (0.3%) participants had an eGFR < 60 mL/min/1.73 m^2^ in this study, both categories were combined into “decreased eGFR” in the following analysis [20].

The World Health Organization has established obesity diagnostic criteria for Asian people, and obesity was defined as a BMI of 25 kg/m^2^ or higher [21]. Previous research has confirmed the validity of this definition [22,23]. Metabolic abnormality was defined as an unhealthy metabolism if at least one of the following was present: (1) systolic blood pressure (SBP) ≥ 130 mmHg and/or a diastolic blood pressure (DBP) ≥ 85 mmHg, or on antihypertensive treatment; (2) high FPG ≥ 5.6 mmol/L, or on medications for diabetes; (3) high TG ≥ 1.7 mmol/L, or on lipid-lowering medications; and (4) low HDL-C < 1.04 mmol/L (if male) [24]. In addition, participants were classified according to the high-sensitivity C-reactive protein (hs-CRP): hs-CRP ≤ 0.01 mg/dL (the median value of this study) and hs-CRP > 0.01 mg/dL [22].

### 2.3. Assessment of Covariates

Age, educational level, smoking status, drinking status, physical activity, and dietary approaches to stop hypertension (DASH) score were considered covariates [25]. Detailed data are available in the Supplementary Materials.

### 2.4. Statistical Analysis

According to the participants’ eGFR status and obesity phenotype, their characteristics were described. The mean and standard deviation of measurements were calculated and assumed a normal distribution. In order to compare between groups, a Student’s *t*-test or an analysis of variance (ANOVA) test was used. To describe and compare continuous variables with non-normal distributions, the Kruskal–Wallis test and median (upper quartile–lower quartile) were used. The *χ*^2^ test method was used to present the data as numbers and percentages for comparing groups.

A logistic regression analysis was conducted to investigate whether decreased eGFR is associated with obesity phenotypes, BMI categories, metabolic abnormality components, or metabolic abnormality number. To evaluate the effects of different obesity phenotypes and inflammation statuses on decreased eGFR, we used logistic regression models as a proxy to assess the combined effect of different obesity phenotypes and inflammation statuses. The MHNO/hs-CRP ≤ 0.01 mg/dL group was defined as the reference. The relative excess risk from interaction, and the proportion attributable to interaction, were used to assess additive interactions between inflammation and obesity on the odds of decreasing eGFR. We measured the relationship between obesity phenotype and decreased eGFR and found that this relationship remained reliable with subgroup analysis of the smoking status, drinking status, physical activity, and hs-CRP (mg/dL). We further adjusted these exposures based on multivariate analysis considering the occupational hazards of workers in steel enterprises. Data analysis was performed using SAS V.9.4 (SAS Institute, Cary, NC, USA). Statistical significance was defined as a *p* < 0.05 with two-tailed tests.

## 3. Results

### 3.1. Characterization of Studies

As shown in Table 1, participants were classified based on their eGFR status and demographic, clinical, and lifestyle characteristics. A total of 6309 participants were included in the present study, with a mean age of 44.64 years and an average eGFR of 101.37 mL/min/1.73 m^2^. Overall, current smokers, current drinkers, obese workers, or those with high blood pressure, high TG, high total cholesterol (TC), high low-density lipoprotein cholesterol (LDL-C), high hs-CRP, and low HDL-C are more likely to have a decreased eGFR (Table 1). Obesity accounted for 56.16% of the total population. The prevalence rates of MHNO, MUNO, MHO, and MUO were 7.32% (*n* = 462), 36.52% (*n* = 2304), 3.57% (*n* = 225), and 52.59 (*n* = 3318), respectively. In addition, the prevalence of different obesity phenotypes showed age differences (Supplementary Table S1).

### 3.2. Relationship among MHO, eGFR, and hs-CRP

The prevalence rate of decreased eGFR was 9.31% (43/462) in the MHNO group, 12.98% (299/2304) in the MUNO group, 16.00% (36/225) in the MHO group, and 17.33% (575/3318) in the MUO group. As shown in Table 2, MHO (OR = 1.95; 95% CI: 1.21 to 3.21) and MUO (OR = 1.70; 95% CI: 1.23 to 2.41) were significantly associated with decreased eGFR compared to MHNO controls. There was no difference in the prevalence rate of decreased eGFR between MUNO and MHNO (Table 2). Cross-classification analysis revealed that in the MHO/hs-CRP > 0.01 mg/dL group, the OR was 2.17 (95% CI: 1.17 to 4.02) for decreased eGFR with the MHNO/hs-CRP ≤ 0.01 mg/dL group as the reference. Each obesity phenotype with the hs-CRP ≤ 0.01 mg/dL was not associated with the decreased eGFR (Table 3).

It is well known that decreased eGFR has been linked to inflammation, and it is likely that exposure to obesity also directly affects inflammation, which could lead to a potential interaction between obesity and hs-CRP. Significant additive or multiplicative interactions between hs-CRP and obesity in terms of the odds of decreased eGFR were not observed after adjustment for the covariates (Table 4).

The association of BMI categories, metabolic abnormality components, and the number of metabolic abnormalities with decreased eGFR are shown in Supplementary Table S2. It has been observed that obese individuals are at an increased risk of having a decreased eGFR as compared to normal-weight individuals (OR = 1.56; 95% CI: 1.34 to 1.80). High BP (OR = 1.24; 95% CI: 1.07 to 1.44), high TG (OR = 1.30; 95% CI: 1.12–1.50), and low HDL-C (OR = 1.23; 95% CI: 1.02 to 1.48) were associated with decreased eGFR; however, FPG was not. Participants with four abnormal metabolic components were associated with decreased eGFR compared to metabolically healthy participants (OR = 1.51; 95% CI: 1.05 to 2.17) (Supplementary Table S2). We also analyzed the relationship between obesity phenotypes and decreased eGFR through stratified analyses based on hs-CRP, drinking status, smoking status, and physical activity. Stratified analyses revealed a significant association between the obesity phenotypes and decreased eGFR in non-smokers, non-drinkers, hs-CRP > 0.01 mg/dL, and both physical activity groups. No interaction was observed between the variables (all *p* for interaction > 0.05) (Supplementary Table S3). It should be noted that the main occupational hazards, such as hot temperatures, sound, carbon monoxide, and dust, that workers in steel industries might be exposed to are also considered in the Supplementary Materials. We found that after adjusting for these exposures, the results of the multivariate analyses between obesity phenotypes and decreased eGFR and the odds ratios of decreased eGFR across obesity phenotypes and inflammatory statuses remained robust (Supplementary Tables S4 and S5).

## 4. Discussion

The current study confirms that, after adjusting for covariates, the MHO phenotype was significantly associated with early-stage renal dysfunction in male steelworkers compared with the MHNO phenotype. This correlation was further attenuated after adjustment for hs-CRP. We also found that the risk of reduced eGFR in individuals with MHO varied depending on the degree of inflammation. Thus, our data provide further evidence that, even in metabolically healthy conditions, excessive obesity can lead to the development of decreased eGFR, and the association between decreased eGFR and MHO phenotypes may be partially explained by an adverse inflammatory status.

Few previous studies have focused on the role of inflammation and the effect of obesity phenotypes on early-stage renal dysfunction, with most focusing on the relationship between obesity phenotypes and CKD. Furthermore, the relationship between obesity phenotypes and decreased eGFR was inconsistent. Our findings are consistent with a large cohort study conducted on 42,128 adults, which concluded that obesity is a risk factor for kidney function decline regardless of whether metabolic health exists (using a BMI cutoff of 30 kg/m^2^ set by the World Health Organization) [26]. In line with our findings, data from the community-based residents of China using an Asian-specific BMI cutoff of ≥ 25 kg/m^2^ showed a significant association between MHO and CKD, partly explained by levels of inflammation [27]. However, a study in Germany showed that a metabolically healthy abdominal obesity phenotype was not associated with an incidence of decreased eGFR [28]. These varying results may be attributed to a discrepancy in the definition of metabolic status, different BMI or waist circumference applications, and different renal endpoints, but the reasons behind them remain unclear. In this study, the International Diabetes Federation’s diagnostic criteria for metabolic syndrome were used to define metabolic status, bridging the deficit of ≥ 1 metabolic risk factor for metabolic health status in the previous criteria. Our results further suggest that obese individuals should receive special attention, despite a normal metabolic status.

In light of the global epidemic of obesity and obesity-related diseases, a better understanding of the mechanisms controlling how obesity contributes to health risks is necessary. The mechanisms by which obesity causes a decrease in eGFR are not fully understood; however, altered hemodynamics, lipid toxicity, and inflammation might explain this link. In central obesity, activating the renin–angiotensin system may lead to altered hemodynamics. In terms of hemodynamic alterations, obesity causes hypertension and glomerular filtration, ultimately resulting in glomerulosclerotic damage [29]. In addition, excessive TG deposition in glomeruli caused by increased adiposity leads to endothelial dysfunction and vascular damage [30,31]. Apart from this, the other potential pathway is chronic inflammation [32]. When adipose tissue expands, the type and level of adipokines will change, such as lipocalin, leptin, and interleukin (IL)−6, which results in chronic structural and functional changes at the nephron level [33,34]. Furthermore, in previous studies, inflammation measured by hs-CRP predicted the development of decreased eGFR, which decreased as inflammation increased [35]. Therefore, consistent with our results, as assessed by hs-CRP, inflammation might be a key factor in inducing a decrease in eGFR among MHO participants.

As a result of the current study, the correlation between MHO phenotype and decreased eGFR was reduced after adjustment for hs-CRP. Furthermore, in the group of hs-CRP > 0.01 mg/dL (using the median of hs-CRP), MHO and MUO phenotypes were significantly associated with decreased eGFR, but this association was not identified in the group of hs-CRP ≤ 0.01 mg/dL. In contrast, MUNO did not associate with decreased eGFR, irrespective of hs-CRP levels. A complementary finding of this study was that when both inflammation and obesity phenotypes were considered, significant odds of decreased eGFR were limited to participants with hs-CRP > 0.01 mg/dL and an MHO or MUO phenotype. In addition, we added the results of the interaction study considering the possible potential interaction between hs-CRP and obesity [36]. However, no link between inflammation and obesity was observed. This study indicates that the inclusion of inflammation into the study of obesity phenotypes with early-stage renal dysfunction would provide more accurate information for health care providers to predict the risk of decreased eGFR.

Our study has several significant strengths, including detailed lifestyle information and larger sample size. To the best of our knowledge, this study is a novel attempt to investigate the effect of inflammation on the relationship between MHO and decreased eGFR among male steelworkers. However, several limitations of this study also need to be considered. First, eGFR was calculated using the CKD-EPI equation, which is primarily affected by creatinine level and can over- or underestimate the actual GFR. Second, although the information on physical activity was assessed quantitatively, it is difficult to identify the difference in the physical activity between the two groups because of the high labor intensity of the steelworkers. However, other studies have shown that physical activity has beneficial effects on health status [37,38]. Third, BMI was used to classify obesity, but BMI cannot distinguish between adipose tissue and lean tissue. Nevertheless, BMI has been shown to be a good indicator of obesity and is considered to be closely related to fat mass [39]. Fourth, we lack data on inflammatory markers other than hs-CRP. Lastly, our study was conducted among male steelworkers in northern China, so the generalizability of the results is limited.

## 5. Conclusions

In conclusion, our data provide further evidence that MHO is not a relatively healthy state; male steelworkers with MHO are at risk of decreased eGFR compared to those without obesity and metabolic abnormalities. Inflammation partially explains this increased risk. Future large-scale prospective studies are needed as a confirmation of these findings.

## Figures and Tables

**Table 1:** Basic characteristics by eGFR status.

Variables	Overall	Non-Decreased eGFR	Decreased eGFR	*p* Value
	*n* = 6309	*n* = 5356	*n* = 953	
Age (years), mean (SD)	44.64 (8.31)	43.97 (8.43)	48.40 (6.46)	<0.001
Age (years), *n* (%)				<0.001
22–29	329 (5.21)	319 (5.96)	10 (1.05)	
30–39	1478 (23.43)	1383 (25.82)	95 (9.97)	
40–49	2369 (37.55)	2006 (37.45)	363 (38.09)	
50–60	2133 (33.81)	1648 (30.77)	485 (50.89)	
Education level, *n* (%)				<0.001
High school or below	4860 (77.03)	4048 (75.58)	812 (85.20)	
University or college	1449 (22.97)	1308 (24.42)	141 (14.80)	
BMI (kg/m^2^), mean (SD)	25.77 (3.70)	25.69 (3.75)	26.26 (3.35)	<0.001
BMI (kg/m^2^), *n* (%)				<0.001
< 25	2766 (43.84)	2424 (45.26)	342 (35.89)	
≥ 25	3543 (56.16)	2932 (54.74)	611 (64.11)	
Lifestyle factors				
Smoking status, *n* (%)				0.009
Never	2313 (36.66)	1956 (36.52)	357 (37.46)	
Ever	458 (7.26)	368 (6.87)	90 (9.44)	
Current	3538 (56.08)	3032 (56.61)	506 (53.10)	
Drinking status, *n* (%)				0.004
Never	3422 (54.22)	2900 (54.14)	522 (54.77)	
Ever	244 (3.87)	190 (3.55)	54 (5.67)	
Current	2643 (41.89)	2266 (42.31)	377 (39.56)	
Physical activity, *n* (%)				0.891
Low	1178 (18.67)	1005 (18.76)	173 (18.15)	
Moderate	3041 (48.20)	2581 (48.19)	460 (48.20)	
High	2090 (33.13)	1770 (33.05)	320 (33.58)	
DASH score, mean (SD)	26.92 (2.74)	26.90 (2.72)	27.01 (2.90)	0.259
Blood pressure (mmHg)				
SBP, mean (SD)	129.57 (15.88)	129.10 (15.63)	132.20 (16.95)	<0.001
DBP, mean (SD)	82.95 (10.34)	82.56 (10.18)	85.15 (10.94)	<0.001
Blood biochemistry (mmol/L)				
TC, mean (SD)	5.14 (0.97)	5.13 (0.97)	5.24 (0.96)	0.001
TG, median (IQR)	1.33 (0.92–1.99)	1.31 (0.90–1.98)	1.43 (1.00–2.06)	<0.001
HDL-C, mean (SD)	1.28 (0.32)	1.29 (0.33)	1.25(0.29)	0.002
LDL-C, mean (SD)	3.23 (0.86)	3.21 (0.86)	3.35 (0.86)	<0.001
FPG, mean (SD)	6.11 (1.35)	6.11 (1.38)	6.13 (1.16)	0.599
hs-CRP (mg/dL), median (IQR)	0.01 (0.00–0.07)	0.01 (0.00–0.06)	0.02 (0.00–0.08)	0.006

SBP, systolic blood pressure; DBP, diastolic blood pressure.

**Table 2:** Odds ratios (95% CIs) for decreased eGFR in different obesity phenotypes.

	MHNO	MUNO	MHO	MUO
Cases/number (%)	43/462 (9.31)	299/2304 (12.98)	36/225 (16.00)	575/3318 (17.33)
Model 1	1.00	1.45 (1.04 to 2.04)	1.86 (1.15 to 2.98)	2.04 (1.47 to 2.83)
Model 2	1.00	1.12 (0.79 to 1.58)	1.98 (1.22 to 3.23)	1.70 (1.22 to 2.40)
Model 3	1.00	1.14 (0.81 to 1.61)	1.97 (1.21 to 3.22)	1.74 (1.25 to 2.43)
Model 4	1.00	1.13 (0.80 to 1.60)	1.95 (1.20 to 3.17)	1.70 (1.21 to 2.38)

Model 1: unadjusted. Model 2: model 1 + adjusted for age (23–29, 30–39, 40–49, 50–60). Model 3: model 2 + adjusted for educational level (high school or below, university or college), drinking status (never, ever, current), smoking status (never, ever, current), physical activity (low, moderate, high), DASH score (continuous variable). Model 4: model 3 + adjusted for hs-CRP (≤0.01, >0.01).

**Table 3:** Odds ratios (with 95% CIs) of decreased eGFR for different obesity phenotypes and inflammation status.

hs-CRP (mg/dL)	Obesity Phenotype	Decreased eGFR		OR (95% CI)
		No, (*n* (%))	Yes, (*n* (%))	
≤0.01	MHNO	298 (5.56)	36 (3.78)	1.00
≤0.01	MUNO	1253 (23.39)	181 (18.99)	0.94 (0.64 to 1.39)
≤0.01	MHO	103 (1.92)	16 (1.68)	1.28 (0.67 to 2.44)
≤0.01	MUO	1161 (21.68)	231 (24.24)	1.36 (0.93 to 1.99)
>0.01	MHNO	121 (2.26)	7 (0.73)	0.46 (0.20 to 1.08)
>0.01	MUNO	752 (14.04)	118 (12.38)	0.98 (0.65 to 1.47)
>0.01	MHO	86 (1.61)	20 (2.10)	2.17 (1.17 to 4.02)
>0.01	MUO	1582 (29.54)	344 (36.10)	1.53 (1.06 to 2.23)

Adjusted for age (23–29, 30–39, 40–49, 50–60), educational level (high school or below, university or college), drinking status (never, ever, current), smoking status (never, ever, current), physical activity (low, moderate, high), and DASH score (continuous variable).

**Table 4:** Interactions between hs-CRP and obesity on the odds of decreased eGFR.

Interaction Terms	Total
Additive interaction ^a^	
Relative excess risk due to interaction, RERI (95% CI)	0.25 (−0.10 to 0.60)
Attributable proportion due to interaction, AP (95% CI)	0.15 (−0.06 to 0.37)
Synergy index, S (95% CI)	1.65 (0.65 to 4.20)
Multiplicative interaction, OR (95% CI)	1.63 (1.35 to 1.97)

^a^: No biological interaction, RERI and AP are equal to 0, and S is equal to 1. Adjusted for age (23–29, 30–39, 40–49, 50–60), educational level (high school or below, university or college), drinking status (never, ever, current), smoking status (never, ever, current), physical activity (low, moderate, high), DASH score (continuous variable), and metabolic abnormality (no/yes).

## Data Availability

The datasets generated and analyzed during this study are available from the corresponding author upon reasonable request.

## Supplementary Materials

The following supporting information can be downloaded at: https://www.mdpi.com/article/10.3390/ijerph191811764/s1, Table S1: Basic characteristics according to obesity phenotypes; Table S2: Odds ratios (with 95% CIs) of decreased eGFR according to BMI, metabolic abnormality components, and metabolically healthy status; Table S3: Multivariate adjusted odds ratios for the association between obesity phenotypes and decreased eGFR, stratified by smoking status, drinking status, physical activity, and hs-CRP; Table S4: Independent effect of obesity phenotypes on decreased eGFR after further adjustment for the main occupational hazards. Table S5: Odds ratios (with 95% CIs) of decreased eGFR for different obesity phenotypes and inflammation status after further adjustment for the main occupational hazards. References [40,41,42,43,44,45,46,47,48,49,50] are cited in the Supplementary Materials.
