# Supplementary material for: Association of Metabolically Healthy Obesity and Glomerular Filtration Rate among Male Steelworkers in North China
Source: Int J Environ Res Public Health. 2022 Sep 18;19(18):11764. doi: 10.3390/ijerph191811764 (PMC9517549; doi:10.3390/ijerph191811764)
Supplement: Supplementary file 1 [file ijerph-19-11764-s001.zip › ijerph-1841396-supplementary.pdf]

Supplementary file

Association between neck circumference and subclinical atherosclerosis among Chinese steelworkers: a cross-sectional survey

Assessment of covariates

Standard study protocols were used to train qualified physicians and nurses prior to this survey. Height and weight were measured three times each. The participants stood upright and barefoot in light clothes.

Blood pressure measurements were performed three times five-minute intervals using an electronic sphygmomanometer (OMRON, HBP-1100, China), and the participants were required to rest for more than ten minutes. Finally, the mean was obtained for analysis.

Participants were required to fast overnight before the health examination and blood collection. Participants' anterior elbow vein blood was collected and centrifuged at room temperature (3000 r/min, 15 minutes) immediately. All blood samples were tested in the central laboratory of Tangshan Hongci Hospital Laboratory using automatic biochemical analysers (Mindrary, BS-800, China) within four hours.

Smoking status was evaluated from self-reported information, mainly including the age at starting smoking and the number of cigarettes consumed per day, and was divided into never smokers (who had never smoked in their lifetime); ever smokers (who had quit cigarettes earlier than 12 months before) and current smokers (who had regularly consumed ≥1 cigarette/day for at least the past 12 months)[1].

Drinking status was evaluated from self-reported information, mainly including the amount and frequency of alcohol consumed per week and was divided into never drinkers (never or almost never drank alcohol in the past 12 months and had not drunk in most weeks in any past year); ever drinkers (did not drink alcohol in most weeks in the past 12 months but did so in some past year(s)) and current drinkers (drank alcohol usually at least once a week over the past 12 months)[2].

The calculation of metabolic equivalents was based on the International Physical Activity Questionnaire (IPAQ)[3]. The workers with metabolic equivalent task (MET) [min/week] values < 600, 600-3000 and > 3000 were classified as having a low, moderate, and high level of physical activity respectively.

Dietary patterns were assessed based on the DASH diet score[4]. Dietary patterns were assessed based on the DASH diet score, which was based on eight foods and nutrients that were either emphasized or deemphasized in the DASH-style diet[5]. Each component was scored from 1 to 5 points according to fifths of intake, with 5 being the best score for higher intake of vegetables, fruits, nuts and legumes, whole grains, and low fat dairy products and for lower intake of sugar sweetened drinks, red and processed meats, and sodium.

Assessment of main occupational hazards

Exposure to dust was defined as workers who may be exposed to productive dust (inorganic dust, organic dust or mixed dust) during production (GBZ/T 229.1–2010). The total dust in the air of workplace was collected at the breathing zone with a filter membrane, and it's concentration was calculated based on the increased weight of the filter membrane and the amount of gas collected. When the dust concentration in the air ≤50 mg/m^3, a filter membrane with a diameter of 37mm or 40mm was used, otherwise a filter membrane with a diameter of 75mm would be used (GBZ/T 192.1–2007)[6].

Exposure to high temperature (heat stress work) was defined as the average wet-bulb globe temperature (WBGT) index of the workplace is equal or greater than 25℃ in the process of production (GBZ 2.2–2007)[7]. The WBGT index was measured by black-wet bulb globe thermometer. If there was no productive heat source in the workplace, three measuring points were selected to take the average value of WBGT index, while where there was a productive heat source, 3 to 5 measuring points were selected to take the average value of WBGT index. If the workplace was isolated into different thermal or ventilated environment, 2 measuring points were selected to take

the average value of WBGT index (GBZ/T 189.7–2007)[8].

Exposure to industrial toxicant was defined as workers who may be exposed to a variety of harmful chemicals (the toxicant specifically refers to carbon monoxide in this population) during production (GBZ/T 229.2–2010)[9]. Carbon monoxide or carbon dioxide in the air of workplace was pumped into the Non-Dispersive Infrared-Ray (NDIR) analyzer and selectively absorbs their infrared rays. The concentration of carbon monoxide was determined according to the absorption value (GBZ/T 160.28–2004)[10].

Exposure to noise was defined as workers who exposed to a noisy environment where the 8h/d or 40h/week equivalent A-weighted sound pressure level is ≥80dB, which may be harmful to health and hearing (GBZ/T 229.4–2012)[11]. The workplace production noise was measured by a sound level meter. If the distribution of sound field in the workplace was uniform (between-field difference of A-sound levels were less than 3dB(A)), three measuring points were selected to take the average value, otherwise it should be divided into several sound level areas. In each sound field, two measuring points were selected to take the average value (GBZ/T 189.8–2007)[12].

1. Li, X.; Cui, S.; Wu, J.; Wang, L.; Yuan, J. Job category differences in the prevalence and associated factors of insomnia in steel workers in China. *Int. J. Occup. Med. Environ. Health* **2020**, *33*, 215–233.
2. Millwood, I.Y.; Walters, R.G.; Mei, X.W.; Guo, Y.; Yang, L.; Bian, Z.; Bennett, D.A.; Chen, Y.; Dong, C.; Hu, R.; et al: Conventional and genetic evidence on alcohol and vascular disease aetiology: a prospective study of 500 000 men and women in China. *Lancet* **2019**, *393*, 1831–1842.
3. Celis-Morales, C.A.; Perez-Bravo, F.; Ibañez, L.; Salas, C.; Bailey, M.E.S.; Gill, J.M.R. Objective vs. self-reported physical activity and sedentary time: effects of measurement method on relationships with risk biomarkers. *PloS ONE* **2012**, *7*, e36345.
4. Maskarinec, G.; Lim, U.; Jacobs, S.; Monroe, K.R.; Ernst, T.; Buchthal, S.D.; Shepherd, J.A.; Wilkens, L.R.; Marchand, L.L.; Boushey, C.J. Diet Quality in Midadulthood Predicts Visceral Adiposity and Liver Fatness in Older Ages: The Multiethnic Cohort Study. *Obesity* **2017**, *25*, 1442–1450.
5. Fung, T.T.; Chiuve, S.E.; McCullough, M.L.; Rexrode, K.M.; Logroscino, G.; Hu, F.B. Adherence to a DASH-style diet and risk of coronary heart disease and stroke in women. *Arch. Intern. Med.* **2008**, *168*, 713–720.
6. Determination of Dust in the Air of Workplace. Part 1: Total Dust Concentration. Available online: http://niohp.chinacdc.cn/zyysjk/zywsbzml/201210/t20121012_70522.htm (accessed on 13 July 2022).
7. Occupational Exposure Limits for Hazardous Agents in the Workplace. Part 2: Physical Agents. Available online: http://niohp.chinacdc.cn/zyysjk/zywsbzml/201303/t20130329_79199.htm (accessed on 13 July 2022).
8. Measurement of Physical Agents in Workplace. Part 7: Heat Stress. Available online: http://niohp.chinacdc.cn/zyysjk/zywsbzml/201210/t20121012_70527.htm (accessed on 13 July 2022).
9. Classification of Occupational Hazards at Workplaces. Part 2: Occupational Exposure to Chemicals. Available online: http://niohp.chinacdc.cn/zyysjk/zywsbzml/201210/t20121012_70489.htm (accessed on 13 July 2022).
10. Methods for Determination of Inorganic Carbon Compounds in the Air of Workplace. Available online: http://niohp.chinacdc.cn/zyysjk/zywsbzml/201210/t20121015_70624.htm (accessed on 13 July 2022).
11. Classification of Occupational Hazards at Workplaces. Part 4: Occupational Exposure to Noise. Available online: http://niohp.chinacdc.cn/zyysjk/zywsbzml/201307/t20130715_84934.htm (accessed on 13 July 2022).
12. Measurement of Physical Agents in Workplace. Part 8: Noise. Available online: http://niohp.chinacdc.cn/zyysjk/zywsbzml/201210/t20121012_70526.htm (accessed on 13 July 2022).

Table of contents

Supplementary Table S1. Basic characteristics according to obesity phenotypes.

Variables	Total	MHNO	MUNO	MHO	MUO	*P* value
	n=6309	*n*=462	*n*=2304	*n*=225	*n*=3318	
Age (years), mean (SD)	44.64 (8.31)	41.28 (9.24)	45.71 (8.25)	41.00 (8.61)	44.62 (8.01)	<0.001
hs-CRP (mg/dL), median (IQR)	0.01 (0.00–0.07)	0.00 (0.00–0.02)	0.01 (0.00–0.04)	0.01 (0.00–0.05)	0.02 (0.00–0.10)	<0.001
DASH score, mean (SD)	26.92 (2.74)	26.86 (2.61)	27.01 (2.75)	26.89 (2.65)	26.86 (2.76)	0.730
eGFR (mL/min/1.73 m2), mean (SD)	101.37 (11.23)	104.87 (10.55)	101.59 (10.82)	102.93 (11.82)	100.63 (11.46)	<0.001
Age (years), n (%)						<0.001
22–29	329 (5.21)	70 (15.15)	101 (4.38)	29 (12.89)	129 (3.89)	
30–39	1478 (23.43)	129 (27.92)	466 (20.23)	72 (32.00)	811 (24.44)	
40–49	2369 (37.55)	157 (33.98)	832 (36.11)	81 (36.00)	1299 (39.15)	
50–60	2133 (33.81)	106 (22.94)	905 (39.28)	43 (19.11)	1079 (32.52)	
Education level, n (%)						<0.001
High school or below	4860 (77.03)	304 (65.80)	1847 (80.16)	150 (66.67)	2559 (77.12)	
University or college	1449 (22.97)	158 (34.20)	457 (19.84)	75 (33.33)	759 (22.88)	
Smoking status, n (%)						0.107
Never	2313 (36.66)	183 (39.61)	852 (36.98)	88 (39.11)	1190 (35.86)	
Ever	458 (7.26)	18 (3.90)	171 (7.42)	17 (7.56)	252 (7.59)	
Current	3538 (56.08)	261 (56.49)	1281 (55.60)	120 (53.33)	1876 (56.54)	
Drinking status, n (%)						<0.001
Never	3422 (54.24)	302 (65.37)	1212 (52.60)	141 (62.67)	1767 (53.25)	
Ever	244 (3.87)	10 (2.16)	98 (4.25)	12 (5.33)	124 (3.74)	
Current	2643 (41.89)	150 (32.47)	994 (43.14)	72 (32.00)	1427 (43.01)	
Physical activity, n (%)						0.017
Low	1178 (18.67)	70 (15.15)	463 (20.10)	37 (16.44)	608 (18.32)	
Moderate	3041 (48.20)	238 (51.52)	1128 (48.96)	117 (52.00)	1558 (46.96)	
High	2090 (33.13)	154 (33.33)	713 (30.95)	71 (31.56)	1152 (34.72)	
eGFR status						<0.001
Non-Decreased	5356 (84.89)	419 (90.69)	2005 (87.02)	189 (84.00)	2743 (82.67)	
Decreased	953 (15.11)	43 (9.31)	299 (12.98)	36 (16.00)	575 (17.33)	

Supplementary Table S2. Odds ratios (with 95% CIs) of morbidity decreased eGFR according to BMI, metabolic abnormality components, and metabolically healthy status.

	Model 1	Model 2	Model 3	Model 4
BMI (kg/m2)				
<25	1.00	1.00	1.00	1.00
≥25	1.48(1.28 to 1.70)	1.56 (1.35 to 1.80)	1.56 (1.35 to 1.81)	1.56 (1.34 to 1.80)
Metabolic abnormality components				
BP	1.44 (1.25 to 1.67)	1.22 (1.05 to 1.42)	1.24 (1.07 to 1.45)	1.24 (1.07 to 1.44)
FPG	1.28 (1.10 to 1.50)	1.05 (0.90 to 1.23)	1.06 (0.90 to 1.24)	1.05 (0.90 to 1.24)
TG	1.24 (1.07 to 1.43)	1.28 (1.10 to 1.47)	1.30 (1.12 to 1.50)	1.30 (1.12 to 1.50)
HDL-C	1.12 (0.93 to 1.34)	1.26 (1.05 to 1.52)	1.24 (1.03 to 1.50)	1.23 (1.02 to 1.48)
Metabolic abnormality number				
0	1.00	1.00	1.00	1.00
1	1.05 (0.80 to 1.38)	0.88 (0.67 to 1.17)	0.89 (0.68 to 1.19)	0.89 (0.67 to 1.18)
2	1.55 (1.19 to 2.00)	1.19 (0.91 to 1.55)	1.21 (0.93 to 1.58)	1.20 (0.92 to 1.57)
3	1.64 (1.25 to 2.17)	1.27 (0.96 to 1.69)	1.31 (0.99 to 1.75)	1.30 (0.98 to 1.73)
4	1.80 (1.26 to 2.56)	1.50 (1.04 to 2.15)	1.52 (1.06 to 2.19)	1.51 (1.05 to 2.17)

Model 1 unadjusted. Model 2 was further adjusted for age (23–29, 30–39, 40–49, 50–60). Model 3 was further adjusted for educational level (high school or below, university or college), smoking status (never, ever, current), drinking status (never, ever, current), physical activity (low, moderate, high), DASH score (continuous variable). Model 4 was further adjusted for hs-CRP (≤0.01, >0.01).

Supplementary Table S3. Multivariate adjusted odds ratios for the association between obesity phenotypes and decreased eGFR, stratified by smoking status, drinking status, physical activity, and hs-CRP.

	**MHNO**	**MUNO**	**MHO**	**MUO**	***p* for Interaction**
Smoking status					0.440
No	1.00	1.29 (0.75 to 2.21)	2.39 (1.15 to 4.96)	2.11 (1.25 to 3.58)	
Yes	1.00	1.05 (0.67 to 1.64)	1.67 (0.86 to 3.24)	1.50 (0.97 to 2.32)	
Drinking status					0.350
No	1.00	1.50 (0.96 to 2.34)	2.20 (1.20 to 4.11)	2.25 (1.46 to 3.47)	
Yes	1.00	0.73 (0.42 to 1.25)	1.62 (0.73 to 3.62)	1.12 (0.67 to 1.90)	
Physical activity					0.505
Low or moderate	1.00	0.97 (0.65 to 1.47)	1.51 (0.83 to 2.77)	1.50 (1.01 to 2.23)	
High	1.00	1.60 (0.84 to 3.04)	3.38 (1.47 to 7.91)	2.39 (1.29 to 4.44)	
hs-CRP (mg/dL)					0.076
≤0.01	1.00	0.97 (0.63 to 1.39)	1.28 (0.67 to 2.44)	1.35 (0.92 to 1.98)	
>0.01	1.00	2.14 (0.97 to 4.76)	4.70 (1.87 to 11.85)	3.38 (1.55 to 7.38)	

Adjusted for age (23–29, 30–39, 40–49, 50–60), educational level (high school or below, university or college), smoking status (never, ever, current), drinking status (never, ever, current), physical activity (low, moderate, high), DASH score (continuous variable) and hs-CRP (≤0.01, >0.01).

Supplementary Table S4. Independent effect of obesity phenotypes on decreased eGFR after further adjustment for the main occupational hazards.

Obesity phenotype	Decreased eGFR No, (n (%))	Decreased eGFR Yes, (n (%))	OR (95% CI)
MHNO	419 (7.28)	43 (4.51)	1.00
MUNO	2005 (37.43)	299 (31.37)	1.13 (0.80 to 1.60)
MHO	189 (3.53)	36 (3.78)	1.97 (1.21 to 3.20)
MUO	2743 (51.21)	575 (60.34)	1.71 (1.23 to 2.39)

Adjusted for age (23–29, 30–39, 40–49, 50–60), educational level (high school or below, university or college), smoking status (never, ever, current), drinking status (never, ever, current), physical activity (low, moderate, high), DASH score (continuous variable), hs-CRP (≤0.01, >0.01), dust exposure (no/yes), heat stress exposure (no/yes), noise exposure (no/yes), and carbon monoxide exposure (no/yes).

Supplementary Table S5. Odds ratios (with 95% CIs) of decreased eGFR for different obesity phenotypes and inflammation status after further adjustment for the main occupational hazards.

hs-CRP (mg/dL)	Obesity phenotype	Decreased eGFR No, (*n* (%))	Decreased eGFR Yes, (*n* (%))	OR (95% CI)
≤0.01	MHNO	298 (5.56)	36 (3.78)	1.00
≤0.01	MUNO	1253 (23.39)	181 (18.99)	0.94 (0.64 to 1.39)
≤0.01	MHO	103 (1.92)	16 (1.68)	1.28 (0.67 to 2.44)
≤0.01	MUO	1161 (21.68)	231 (24.24)	1.35 (0.92 to 1.98)
>0.01	MHNO	121 (2.26)	7 (0.73)	0.46 (0.20 to 1.08)
>0.01	MUNO	752 (14.04)	118 (12.38)	0.98 (0.65 to 1.47)
>0.01	MHO	86 (1.61)	20 (2.10)	2.17 (1.17 to 4.03)
>0.01	MUO	1582 (29.54)	344 (36.10)	1.53 (1.05 to 2.22)

Adjusted for age (23–29, 30–39, 40–49, 50–60), educational level (high school or below, university or college), drinking status (never, ever, current), smoking status (never, ever, current), physical activity (low, moderate, high), DASH score (continuous variable), dust exposure (no/yes), heat stress exposure (no/yes), noise exposure (no/yes), and carbon monoxide exposure (no/yes).
